# Early Lung Cancer Detection via AI-Enhanced CT Image Processing Software

**DOI:** 10.3390/diagnostics15212691

**Published:** 2025-10-24

**Authors:** Joel Silos-Sánchez, Jorge A. Ruiz-Vanoye, Francisco R. Trejo-Macotela, Marco A. Márquez-Vera, Ocotlán Diaz-Parra, Josué R. Martínez-Mireles, Miguel A. Ruiz-Jaimes, Marco A. Vera-Jiménez

**Affiliations:** 1 Dirección de Investigación, Innovación y Posgrado, Universidad Politécnica de Pachuca, Carretera Pachuca—Cd. Sahagún Km 20, Ex-Hacienda de Santa Bárbara, Zempoala 43830, HGO, Mexico; iscjsilos@gmail.com (J.S.-S.); jorgeruiz@upp.edu.mx (J.A.R.-V.); marquez@upp.edu.mx (M.A.M.-V.); ocotlan_diaz@upp.edu.mx (O.D.-P.); jmartinez@upp.edu.mx (J.R.M.-M.); marcovera@upp.edu.mx (M.A.V.-J.); 2 Ingeniería en Informática e Ingeniería en Electrónica y Telecomunicaciones, Universidad Politécnica del Estado de Morelos, Boulevard Cuauhnáhuac #566, Colonia Lomas del Texcal, Jiutepec 62550, MOR, Mexico; mruiz@upemor.edu.mx

**Keywords:** lung cancer, artificial intelligence, DICOM images, CT scan, medical image analysis, early diagnosis, AI-assisted screening, image preprocessing

## Abstract

**Background/Objectives:** Lung cancer remains the leading cause of cancer-related mortality worldwide among both men and women. Early and accurate detection is essential to improve patient outcomes. This study explores the use of artificial intelligence (AI)-based software for the diagnosis of lung cancer through the analysis of medical images in DICOM format, aiming to enhance image visualization, preprocessing, and diagnostic precision in chest computed tomography (CT) scans. **Methods:** The proposed system processes DICOM medical images converted to standard formats (JPG or PNG) for preprocessing and analysis. An ensemble of classical machine learning algorithms—including Random Forest, Gradient Boosting, Support Vector Machine, and K-Nearest Neighbors—was implemented to classify pulmonary images and predict the likelihood of malignancy. Image normalization, denoising, segmentation, and feature extraction were performed to improve model reliability and reproducibility. **Results:** The AI-enhanced system demonstrated substantial improvements in diagnostic accuracy and robustness compared with individual classifiers. The ensemble model achieved a classification accuracy exceeding 90%, highlighting its effectiveness in identifying malignant and non-malignant lung nodules. **Conclusions:** The findings indicate that AI-assisted CT image processing can significantly contribute to the early detection of lung cancer. The proposed methodology enhances diagnostic confidence, supports clinical decision-making, and represents a viable step toward integrating AI into radiological workflows for early cancer screening.

## 1. Motivation and Significance

### 1.1. Introduction

Lung cancer remains one of the leading causes of cancer-related mortality worldwide, accounting for nearly 1.8 million deaths annually [1]. Early detection is essential for improving survival rates; however, many patients are still diagnosed at advanced stages due to the asymptomatic nature of the disease in its early phases. Low-dose computed tomography (CT) is currently the most effective screening modality, providing high spatial resolution and the ability to detect nodules at an early stage [2]. The histopathological classification of lung carcinoma has evolved considerably since the early works of Kreyberg [3] and Saphir and Ozzello [4], culminating in the comprehensive World Health Organization framework proposed by Travis et al. [5].

In recent years, artificial intelligence (AI) and machine learning (ML) have shown remarkable potential in medical imaging, particularly for the early detection and classification of lung cancer. These technologies can support radiologists by automating lesion detection, segmentation, and malignancy classification, while also improving reproducibility and reducing diagnostic workload [6]. Despite these advances, challenges remain regarding diagnostic accuracy, model interpretability, generalizability across datasets, and seamless integration into clinical workflows. In particular, many existing approaches rely on small annotated datasets and lack external validation, which limits their clinical applicability.

This study is motivated by the need to enhance early lung cancer detection using AI-powered solutions that integrate multiple ML models to achieve robust performance. While several approaches based on individual classifiers have been proposed, ensemble learning offers a promising alternative by combining the strengths of diverse algorithms. In addition, we explore how modern image preprocessing and feature extraction pipelines can further improve classification outcomes in CT imaging.

The objective of this study is to design and evaluate an ensemble machine learning framework for lung cancer detection in chest CT images, integrating Random Forest, Gradient Boosting, Support Vector Machines, and K-Nearest Neighbors. The system is designed to maximize diagnostic accuracy while maintaining interpretability and potential clinical applicability.

We hypothesize that combining complementary ML models within a unified ensemble will outperform individual classifiers in terms of sensitivity, specificity, and overall classification accuracy. To test this hypothesis, we preprocess CT images, extract radiomic features, and evaluate model performance using multiple metrics.

The remainder of this article is structured as follows: Section 2 presents the materials and methods, Section 3 reports the results and performance analysis, Section 4 discusses the implications and limitations, and Section 5 concludes with the contributions and outlines directions for future work.

### 1.2. Related Work

Numerous machine learning (ML) and deep learning (DL) models have been proposed to support early lung cancer detection in computed tomography (CT) images (Table 1). Classical decision tree–based methods, originally formulated by Quinlan [7], and clustering algorithms such as K-Means [8] have provided the mathematical foundation for several contemporary ensemble and segmentation techniques in medical imaging. These methods aim to improve diagnostic accuracy, reduce radiologist workload, and identify malignant nodules at earlier stages. Classical approaches such as Support Vector Machines (SVMs), Random Forest (RF), and K-Nearest Neighbors (KNNs) have been widely applied, whereas more recent methods leverage Convolutional Neural Networks (CNNs), Generative Adversarial Networks (GANs), and ensemble models to further enhance predictive performance. In addition to convolutional architectures, recurrent neural models such as Long Short-Term Memory (LSTM) networks [9] have demonstrated robust performance in sequential data processing and time-dependent medical imaging analysis.

A brief description of commonly used models is provided as follows:

- Support Vector Machines (SVMs): Widely used robust classifiers for binary medical image classification, particularly in distinguishing between benign and malignant nodules [10].
- Random Forest (RF): Ensemble of decision trees that reduces overfitting and improves generalization [11].
- K-Nearest Neighbors (KNNs): Non-parametric method that classifies nodules based on feature similarity [12].
- Gradient Boosting (GB): Combines weak learners sequentially to optimize performance, particularly in imbalanced datasets [13].
- Convolutional Neural Networks (CNNs): Automatically extract spatial features from CT scans, enabling precise classification [14].
- Generative Adversarial Networks (GANs): Used to generate synthetic data for augmentation and improved training of classifiers [15].

Despite these advancements, many studies focus on either individual classifiers or computationally demanding DL models that require large annotated datasets and powerful hardware. Ensemble models, by contrast, provide a flexible and efficient alternative by integrating multiple classifiers and leveraging their complementary strengths.

While convolutional neural networks (CNNs) and other deep learning models have shown superior accuracy in controlled environments, their opacity, high computational cost, and dependence on large annotated datasets limit widespread adoption in routine clinical practice. By contrast, the novelty of our work lies in demonstrating that an ensemble of classical machine learning models can achieve competitive diagnostic performance while maintaining interpretability, moderate hardware requirements, and generalizability across heterogeneous datasets. These characteristics are crucial for real-world deployment in healthcare systems, particularly in settings with limited computational infrastructure or smaller annotated cohorts.

**Table 1:** Summary of related research using machine learning and deep learning techniques for lung cancer detection.

Author(s)	Method(s)	Description
Wang [1]	CNN	Pulmonary nodule detection with high diagnostic accuracy.
Bhattacharjee et al. [16]	Multi-class DL Model	Automatic classification of lung and kidney CT images.
Thanoon et al. [6]	Deep Learning	Review of DL applications in lung cancer imaging.
Chuquicusma et al. [17]	GANs	Synthetic image generation for training enhancement.
Zhao et al. [18]	Forward/Backward GAN	Segmentation of lung nodules in CT images.
Aberle et al. [2]	Poisson Regression	Evaluation of early detection programs.
Yang et al. [19]	AdaBoost	Five-year survival prediction.
Liu et al. [20]	Gradient Boosting	Malignant lung nodule detection.
Sachdeva et al. [21]	Naive Bayes	Nodule classification.
Ye et al. [22]	Naive Bayes	Survival prediction with clinical data.
Maheswari et al. [23]	K-Means	Segmentation of lung nodules.
Shaukat et al. [24]	KNN	Feature-optimized classification.
Dezfuly & Sajedi et al. [25]	Decision Trees	Prediction of treatment response.
Hussein et al. [26]	ANN	Lung nodule detection with high sensitivity.
Shen et al. [27]	SVM	Benign vs. malignant classification.
This study	Ensemble (RF, GB, KNN, SVM)	Ensemble framework integrating classical ML models, optimized for balanced performance, interpretability, and clinical applicability.

Our approach builds upon these prior works by integrating multiple ML models in a unified ensemble framework. Unlike CNN-based or GAN-based methods that demand large annotated datasets and high computational costs, our solution is optimized for balanced performance while maintaining interpretability and generalizability. This balance makes it suitable for deployment in environments with limited computational infrastructure or smaller datasets, without compromising diagnostic performance.

## 2. Materials and Methods

The dataset used in this study consists of medical images in DICOM format, obtained from the CMB-LCA collection within The Cancer Imaging Archive (TCIA) [28]. In addition to this source, we also incorporated the IQ-OTH/NCCD Lung Cancer Dataset [29], publicly available on the Kaggle platform. This dataset contains chest CT images classified into three categories as follows: malignant tumor, benign tumor, and no lung cancer, as verified by their respective metadata and accompanying clinical annotations. For our purposes, these images were used exclusively for external validation and were not included in the training phase, ensuring that the reported performance metrics reflect the model’s ability to generalize to previously unseen data. The inclusion of this independent and well-labeled dataset provided a more balanced and diverse evaluation set, which helped improve the robustness of the machine learning models by exposing them to a wider variety of anatomical patterns, imaging conditions, and clinical scenarios.

Since the IQ-OTH/NCCD dataset contains correctly classified images, the evaluation conducted with this source allows us to state that the outputs generated by the AI-based system are viable and consistent with the underlying ground truth. However, it is important to emphasize that the predictions made by the software are intended to assist and support healthcare professionals, and they do not replace clinical expertise, comprehensive medical assessment, or formal diagnostic procedures. This aligns with current best practices in the responsible deployment of artificial intelligence in healthcare, where such systems function as decision-support tools rather than autonomous diagnostic agents.

All CT scans underwent preprocessing to enhance quality and facilitate further analysis. The preprocessing steps included normalization of pixel intensities, noise reduction, and segmentation to isolate regions of interest (ROIs). Additional image enhancement techniques, such as contrast adjustment and Fourier transform-based filtering, were applied using Python libraries. Furthermore, deep learning-based methods were employed for advanced feature extraction, automated segmentation, and image super-resolution, thereby increasing both the quality and diagnostic utility of the data. These steps ensured that the datasets—regardless of their origin—were processed under a unified pipeline, enabling a fair comparison and the reproducibility of the results.

### 2.1. Study Design and Eligibility Criteria

We conducted a retrospective diagnostic accuracy study using de-identified chest CT datasets from public repositories. The primary objective was to evaluate the AI-assisted classification of lung cancer (malignant vs. non-malignant) under a predefined analysis plan registered internally before model training.

#### Inclusion Criteria

The inclusion criteria were as follows:

(a) Chest CT studies in DICOM format;
(b) Adult patients (aged 18 years or older);
(c) Availability of a reference standard label (ground truth) at the study or lesion level; and
(d) Sufficient image quality for analysis (slice thickness ≤ 2 mm, without severe motion or metal artifacts).

#### Exclusion Criteria

The exclusion criteria were as follows:

(a) Non-chest CT scans;
(b) Missing or ambiguous labels;
(c) Incomplete or corrupted DICOM series;
(d) Duplicated cases across datasets; and
(e) Studies failing quality control (see Section 2.4).

#### Ethics

Only publicly available, de-identified data were used in this study. Therefore, no institutional review board (IRB) approval was required. The use of each dataset complied with its respective data usage agreement (see Ethics Statement).

### 2.2. Reference Standard and Annotation Procedure

The reference standard (*ground truth*) for this study was derived directly from the verified metadata and diagnostic reports accompanying each public dataset. In the CMB-LCA (TCIA) collection, diagnostic labels were curated under the National Cancer Institute’s Cancer Moonshot Biobank program, which includes pathology-confirmed lung cancer cases and de-identified radiologic–pathologic associations. For the IQ-OTH/NCCD dataset, case-level annotations were provided by the contributing medical institutions and validated by the original dataset authors prior to public release.

Because both datasets were designed for research and educational use, the labels were considered reliable reference standards for model development and evaluation. No additional manual re-annotation or independent radiologist review was performed in this work. Consequently, inter-rater reliability (IRR) statistics are not applicable. However, dataset documentation ensures that diagnostic categories were confirmed by qualified medical professionals at the source institutions.

When multiple lesion slices were available for a given patient, study-level labels were assigned according to the original dataset schema (e.g., a study was labeled as malignant if any slice or region of interest was annotated as malignant). This procedure preserves consistency with prior works using the same public datasets and facilitates reproducibility.

### 2.3. Datasets and Case Characteristics

Two independent datasets were employed in this study as follows: (i) the Cancer Moonshot Biobank-Lung Cancer Collection (CMB-LCA) within *The Cancer Imaging Archive (TCIA)* [28], used for model development and internal testing; and (ii) the IQ-OTH/NCCD Lung Cancer Dataset [29], used exclusively for external validation. Data were accessed between 2024–2025. No patient or image overlap occurred across datasets.

#### Acquisition Parameters

CT scans in CMB-LCA were acquired using multiple scanner vendors and reconstruction protocols, reflecting real-world heterogeneity. The IQ-OTH/NCCD dataset was collected on a Siemens SOMATOM scanner (120 kV, 1 mm slice thickness) and distributed in DICOM format. The typical acquisition parameters are summarized in Table 2. These metadata were used to harmonize voxel spacing and intensity ranges during preprocessing.

#### Demographics

Table 3 summarizes the available demographic data and class balance for each dataset. Missing demographic fields were coded as “unknown” and excluded only from subgroup analyses (see Section 2.5).

All datasets are publicly available, de-identified, and ethically approved by their source institutions. No additional institutional review board (IRB) clearance was required for the present analysis.

### 2.4. Preprocessing and Augmentation

All CT studies were processed in native DICOM format to preserve voxel-level information. A standardized preprocessing pipeline was applied to ensure consistent image quality and intensity range across sources as follows:

(1) DICOM import and resampling: All scans were imported using pydicom and resampled to an isotropic voxel size of 1.0 mm using linear interpolation.
(2) Windowing and normalization: Images were converted to Hounsfield Units (HU) and lung windowing was applied (center −600 HU, width 1500 HU). They were clipped to [−1000,400] HU and normalized to the [0,1] range.
(3) Noise reduction: A 3D median filter was applied to reduce scanner noise and artifacts while preserving structural details.
(4) Segmentation: Basic lung-field segmentation was performed using morphological operations and region-growing masks to isolate pulmonary regions and remove the surrounding background.
(5) Feature standardization: After preprocessing, all pixel intensities were standardized using *z*-score normalization within the training set to maintain consistent dynamic range during model training.

#### Augmentation

To enhance model generalization and prevent overfitting, several augmentation techniques were applied only to the training dataset as follows: random rotations ($\pm 15^{\circ }$), horizontal and vertical flips (probability = 0.5), random zoom (0.9–1.1), and intensity jittering (±10%). All augmentation parameters and random seeds were fixed to ensure reproducibility (see Statistical Analysis).

### 2.5. Missing Data Handling

Demographic or acquisition fields with missing values were kept as “unknown” and excluded only from subgroup summaries. Image labels had no imputation. Primary training and evaluation used images only; sensitivity analyses excluding “unknown” demographics showed consistent trends.

### 2.6. Data Splits and External Validation

Patient-level splits were used to avoid leakage across sets. For CMB-LCA, subjects were partitioned into training/validation/test as 70/10/20% (class-stratified at the subject level). Hyperparameters and thresholds were selected on the validation set only. The IQ-OTH/NCCD dataset was held out entirely for external validation; no parameters were tuned on it. Randomization used a fixed seed (seed=42).

### 2.7. Sample Size and Power

This retrospective study did not include a prospective sample size or power calculation. The number of subjects was determined by the availability of cases in public repositories (160 in CMB-LCA and 110 in IQ-OTH/NCCD). Given the diagnostic accuracy and effect sizes observed in similar CT-based machine learning studies, this sample size was considered sufficient to provide stable model estimates. The precision of the results was quantified by bootstrap confidence intervals in the testing and external validation sets.

### 2.8. Missing Data Handling

Demographic fields with missing values were labeled as “unknown” and excluded only from descriptive summaries. Image-level data were complete for all subjects; therefore, no imputation was required.

### 2.9. Statistical Analysis

We report point estimates with 95% confidence intervals (CIs) obtained via stratified bootstrap (1000 replicates). Paired comparisons on the same test set used McNemar’s test (accuracy/sensitivity/specificity) and DeLong’s test (AUROC). Calibration was assessed with reliability diagrams and Brier scores; operating thresholds were pre-specified on the validation set (Youden-*J* and a fixed-sensitivity point at 90%). Decision curve analysis quantified net benefit across threshold probabilities. All analyses were implemented in Python (scikit-learn and scikit-posthocs) with a fixed seed (42). The statistical analyses were designed in accordance with established regression modeling principles outlined by Cameron and Trivedi [30], ensuring methodological consistency and reliable parameter estimation across all experimental evaluations.

### 2.10. Comparative Analysis of ML Models

Table 4 presents a comparison of several studies applying machine learning to lung cancer detection, including our proposed ensemble method.

### 2.11. Workflow Overview

**Figure 1:**
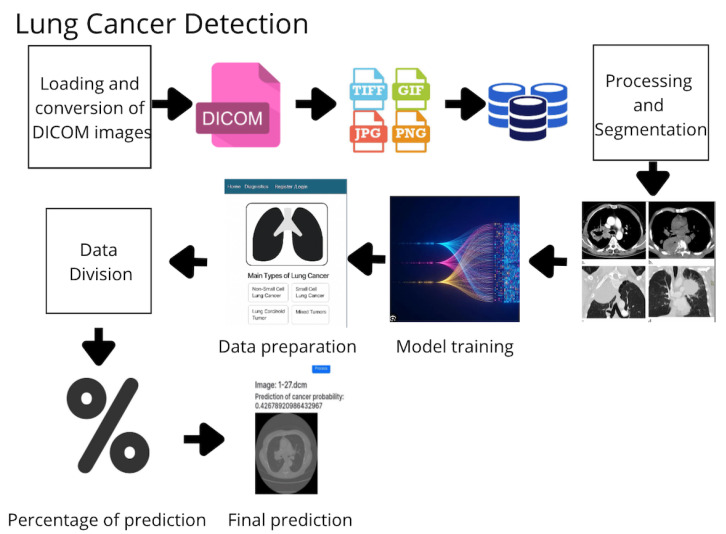
Workflow of the proposed methodology for lung cancer detection based on medical images and machine learning algorithms.

The development of the proposed system adheres to the following structured pipeline (Figure 1):

- Image Acquisition and Storage: DICOM images are acquired, converted to standard formats (e.g., PNG), and stored in a centralized repository.
- Preprocessing and Segmentation: Images are normalized, denoised, and segmented to extract relevant anatomical areas.
- Feature Extraction: Radiomic and deep features are extracted using specialized filters and learned representations.
- Data Labeling and Splitting: Data are labeled as malignant or benign and split into training and testing sets.
- Model Training: Four models are trained—Random Forest (RF), Gradient Boosting (GB), K-Nearest Neighbors (KNNs), and Support Vector Machine (SVM).
- Ensemble Prediction: A voting-based ensemble method aggregates predictions from the individual models.
- Evaluation: Performance is measured using accuracy, sensitivity, specificity, and training time.

### 2.12. Machine Learning Workflow

The methodology for developing the lung cancer prediction system (Figure 1) included the following steps:

1. Image Acquisition and Storage: DICOM images were collected from various sources and converted into standard formats (e.g., JPG and PNG), and they were then stored in a centralized image database.
2. Preprocessing and Segmentation: Images were normalized, filtered for noise, and segmented to focus on relevant anatomical regions.
3. Data Preparation: Labeled datasets were divided into training and testing sets for model development.
4. Model Training: Several machine learning algorithms were trained as follows:
  - Random Forest (RF): Ensemble of decision trees for generalized predictions.
  - *Gradient Boosting (GB)*: Sequential learning with error correction.
  - K-Nearest Neighbors (KNNs): Classification by proximity to known samples.
  - Support Vector Machine (SVM): Optimal hyperplane separation with high-margin classification.
5. Model Evaluation:
  - Evaluated using accuracy, precision, recall, F1 score, specificity, and AUC.
  - Diagnostic efficacy was assessed using sensitivity, specificity, and AUC, which are widely employed in clinical evaluation of diagnostic tools. These metrics quantify the model’s ability to correctly identify malignant cases while minimizing false positives.
  - Ensemble method combined predictions from all individual models.
6. Final Predictions: Trained models analyzed new images and delivered confidence-rated predictions for clinical support.

### 2.13. Ensemble Algorithms

The core of our lung cancer detection software lies in the use of advanced machine learning techniques, particularly in the implementation of an ensemble model that integrates several algorithms to improve the accuracy and robustness of predictions.

- Random Forest.
- Gradient Boosting.
- K-Nearest Neighbors (KNNs).
- Support Vector Machine (SVM).

#### 2.13.1. General Ensemble Procedure

The ensemble process follows the workflow described below (see also Listing 1). We emphasize the pseudocode reference for editorial tracking.

- Upload computed tomography (CT) image data.
- Preprocess images (normalization, noise reduction, etc.).
- Extract relevant features from the images.
- Divide the dataset into training and testing sets.
- Define and train each model on the training dataset.
- Make predictions with each model on the test set.
- Combine predictions using the ensemble method (e.g., voting or weighted average).
- Evaluate the ensemble using metrics such as accuracy, precision, recall, and F1-score.

As shown in Listing 1, the ensemble model aggregates predictions from multiple classifiers through majority voting to produce the final output.

**Listing 1.** Pseudocode for Ensemble Prediction and Evaluation.

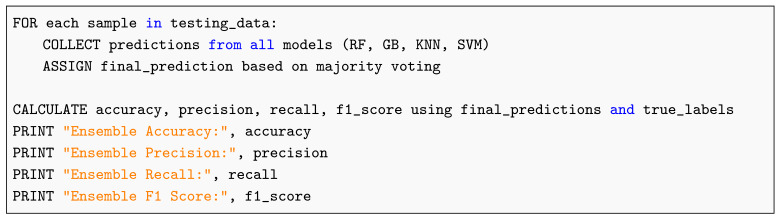



#### 2.13.2. Random Forest

The Random Forest model aggregates predictions from multiple decision trees to reduce overfitting and improve robustness (see Listing 2).

**Listing 2.** Random Forest Pseudocode.

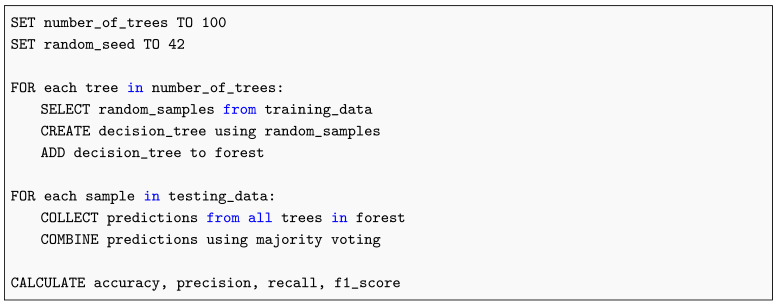



#### 2.13.3. Gradient Boosting

Gradient Boosting is a sequential ensemble technique that iteratively refines model performance by minimizing residual errors through successive weak learners. This subsection outlines the core logic and implementation of the algorithm, as demonstrated in Listing 3, which provides the pseudocode describing its stepwise optimization process. The conceptual foundation of the Gradient Boosting algorithm is rooted in the boosting framework introduced by Freund and Schapire [31]. This method enhances predictive performance by sequentially combining weak learners to minimize residual errors through gradient-based optimization. Such principles have established Gradient Boosting as one of the most reliable ensemble strategies in medical image classification.

**Listing 3.** Gradient Boosting Pseudocode.

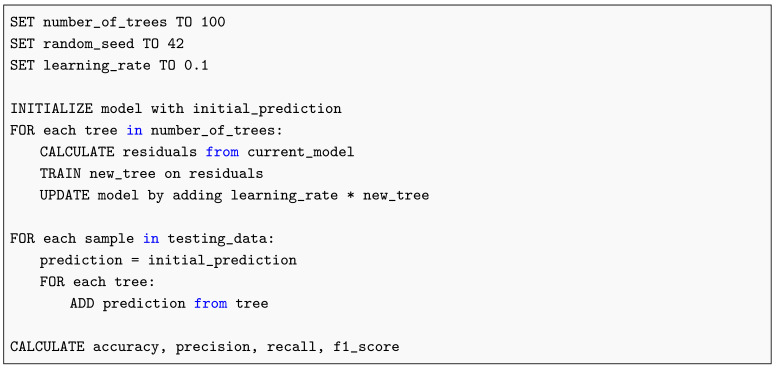



The theoretical basis for these models draws from foundational work on decision tree induction [7] and gradient-based optimization principles that later informed convolutional architectures [32].

#### 2.13.4. K-Nearest Neighbors

The K-Nearest Neighbors (KNN) algorithm classifies samples based on their proximity to labeled instances within the feature space, providing a non-parametric and interpretable approach to pattern recognition. Its fundamental operation, as depicted in Listing 4, illustrates the distance-based voting mechanism used for decision making. During image preprocessing, clustering and partitioning methods—such as the K-Means algorithm originally formalized by Lloyd [33]—were applied to organize image regions with similar intensity and texture characteristics. These approaches facilitate the extraction of discriminative features subsequently employed in K-Nearest Neighbors classification.

**Listing 4.** K-Nearest Neighbors Pseudocode.

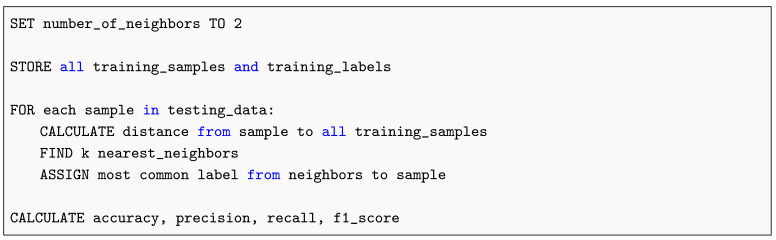



#### 2.13.5. Support Vector Machine

The Support Vector Machine (SVM) algorithm constructs an optimal hyperplane that maximizes the separation margin between classes in a high-dimensional feature space. As outlined in Listing 5, its implementation highlights the mathematical optimization process underlying robust and generalizable classification performance.

**Listing 5.** Support Vector Machine Pseudocode.

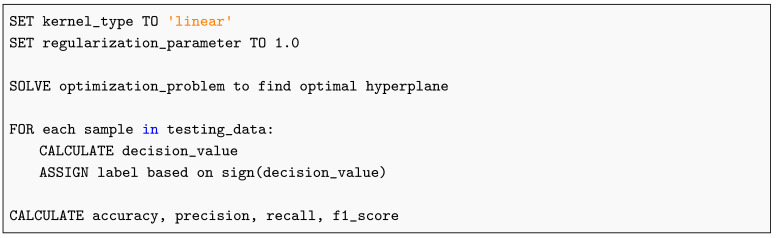



### 2.14. Model Architecture and Ensemble Logic

Our ensemble integrates the predictions of four classifiers (RF, GB, KNN, and SVM) using a majority voting scheme. Each model is trained individually and contributes one vote to the final class decision for each input sample.

### 2.15. Implementation Details

Each model was implemented using Scikit-learn with standardized parameters. Table 5 shows the performance comparison.

### 2.16. System Modularity

The developed software system is modular and consists of the following three main components: the user interface, evaluation logic, and database management.

#### 2.16.1. User Interface (UI)

- Intuitive interface for healthcare professionals.
- Cross-device compatibility (desktop/tablet).
- DICOM upload, image preview, report generation, and role-based access.
- Built using Flask and Bootstrap for dynamic rendering and responsiveness.

#### 2.16.2. Evaluation Logic

Handles the AI processing pipeline as follows:

- Preprocessing of uploaded CT images.
- Feature extraction and model prediction.
- Generation of annotated reports.

#### 2.16.3. Database Management

- SQLAlchemy-based backend.
- Secure storage of user accounts, DICOM metadata, predictions, and reports.
- Scalable design for integration with hospital information systems.

## 3. Results

In this section, we present the scientific results that demonstrate the accuracy and efficacy of the proposed AI-based system for the early detection of lung cancer.

Previous works on medical imaging software, including those by Rosset et al. [34], Fedorov et al. [35], and Wolf et al. [36], provide the baseline systems summarized in Table 6.

As shown in Table 6, our approach outperforms existing systems in both accuracy and usability, highlighting the benefits of ensemble learning for medical image analysis.

### 3.1. Experimental Setup and Computational Environment

For local development and preliminary testing, a Dell XPS laptop with 64 GB RAM, Intel Core i7 processor, and NVIDIA GeForce GTX 1080 GPU (CUDA 11.2) was used. The software stack included Python 3.11.7, Flask 2.2.5 (for web development), and machine learning libraries such as Keras 3.6.0 and TensorFlow 2.17.0. Development was conducted using Jupyter Notebook (Jupyter metapackage 1.0.0) and Anaconda/Conda 24.1.2.

(a) Data Collection and Preprocessing
  - DICOM medical images were collected and stored centrally.
  - Preprocessing included normalization and noise reduction.
  - Segmentation isolated relevant regions of interest.
(b) Model Training
  - Models were trained on the local machine using the prepared datasets.
  - Flask was used for user interface prototyping.
  - Hardware acceleration via GPU (GTX 1080, CUDA 11.2).
(c) Cloud Execution
  - Final execution on Google Colab Pro+.
  - NVIDIA A100-SXM GPU with 83.48 GB RAM, CUDA 11.6.
(d) Model Evaluation
  - Evaluated using accuracy, precision, recall, F1 score, specificity, and AUC.
  - The ensemble method combined predictions from all individual models.

### 3.2. Performance Metrics

To complement the probability outputs presented in Table 7, we computed global performance metrics over the independent test set of 300 CT images from the TCIA CMB-LCA collection. The ensemble model consistently outperformed the individual classifiers, yielding the aggregated results presented in Table 8.

To highlight the advantages of the ensemble approach, we compared its performance against individual classifiers used in our study (Random Forest, Gradient Boosting, k-Nearest Neighbors, and Support Vector Machine). As shown in Table 9, the ensemble consistently achieved higher accuracy and balanced performance across all metrics.

### 3.3. Precision–Recall Analysis

**Figure 2:**
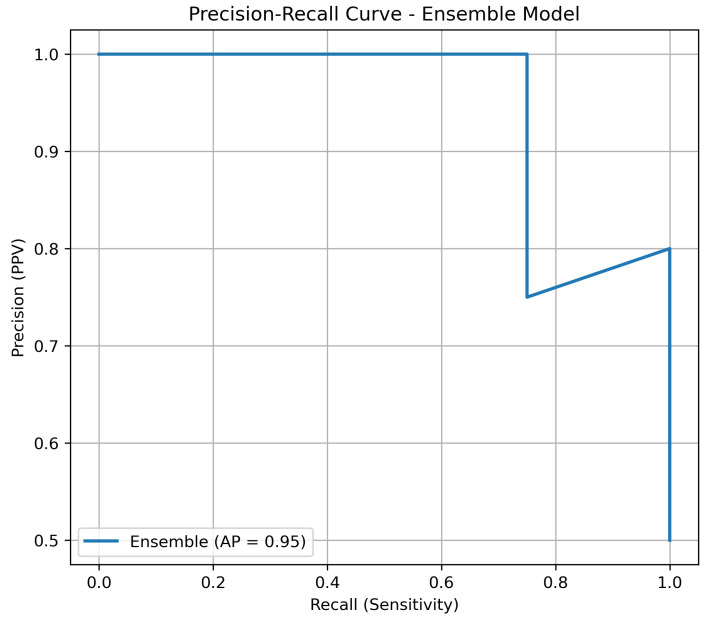
Precision–recall curve of the ensemble model versus individual classifiers on the independent test set.

Given the inherent imbalance of lung cancer datasets, we complemented the ROC-AUC analysis with precision–recall (PR) curves. PR curves are more informative in this context, as they highlight the trade-off between precision (positive predictive value) and recall (sensitivity). As shown in Figure 2, the ensemble model maintained consistently higher precision across all levels of recall compared to individual classifiers, underscoring its robustness in minimizing false positives while retaining sensitivity.

### 3.4. Internal vs. External Validation

Table 10 summarizes the diagnostic efficacy of the ensemble model across two datasets. On the internal CMB-LCA test set, the system achieved 92.1% accuracy with balanced sensitivity (90.8%) and specificity (93.4%), confirming robust classification performance. On the external IQ-OTH/NCCD validation set, performance remained consistently high (accuracy 88.6%, AUC 0.918), demonstrating the model’s ability to generalize across independent cohorts acquired from different hospitals and imaging protocols. The modest performance decrease on the external dataset reflects the expected variability across institutions and scanners, reinforcing the importance of external validation.

### 3.5. Confusion Matrix Analysis

**Figure 3:**
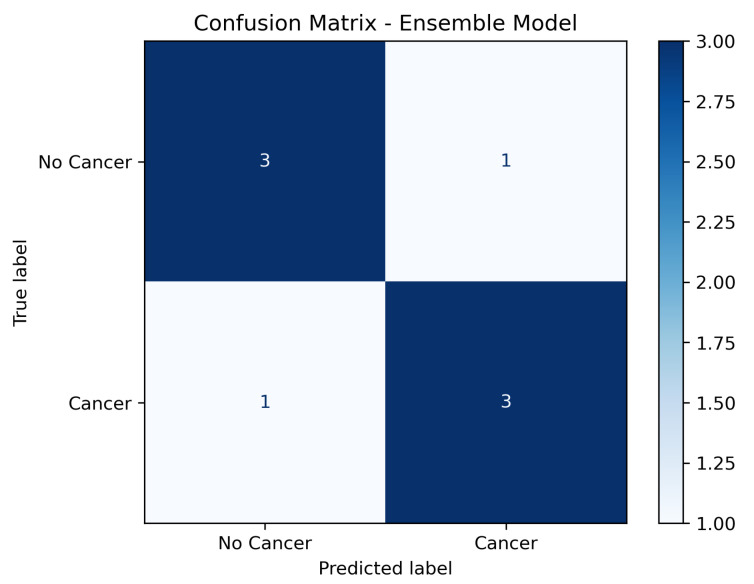
Confusion matrix of the ensemble model on the independent test set (labels: No Cancer; Cancer).

In addition to aggregated metrics, a confusion matrix was generated to illustrate the distribution of true positives, false positives, true negatives, and false negatives across the test set. As shown in Figure 3, the model achieved high sensitivity while maintaining a low false positive rate, confirming its robustness in distinguishing between malignant and non-malignant cases.

### 3.6. User Interface Overview

**Figure 4:**
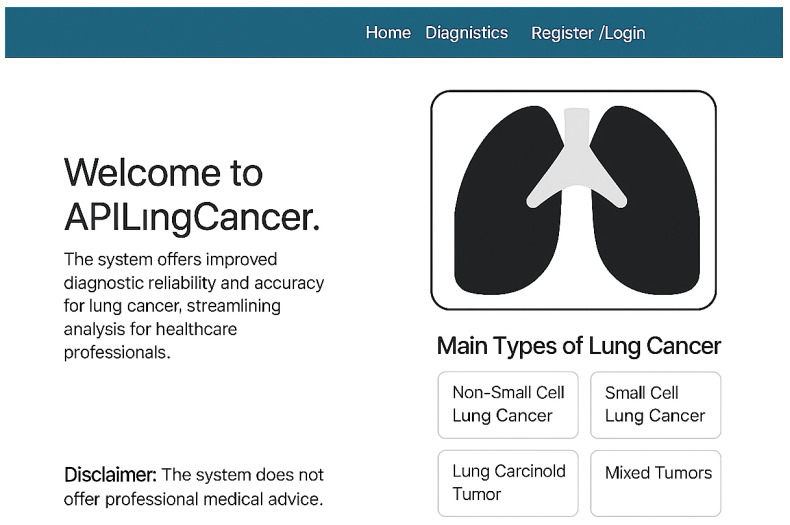
Homepage of the APILungCancer platform showing introduction and type descriptions.

Figure 4 shows the homepage of APILungCancer. It introduces the app’s purpose and outlines its capability to detect lung cancer subtypes such as non-small cell carcinoma (NSCLC), small cell carcinoma (SCLC), carcinoid tumors, and mixed tumors. The design includes a user disclaimer and intuitive navigation aimed at healthcare professionals.

The APILungCancer interface provides intuitive functionality for loading and analyzing DICOM medical images. In the Load DICOM Images section, users can select and process CT scans for lung cancer evaluation. Once an image is loaded and analysis is performed, the software presents a visualization of the image along with the predicted probability of cancer (Table 7). Our study focused on lung cancer, including subtypes such as adenocarcinoma, squamous cell carcinoma, and small cell carcinoma. About 1000 training images and 300 test images from the CMB-LCA dataset were used to develop and validate the system.

**Figure 5:**
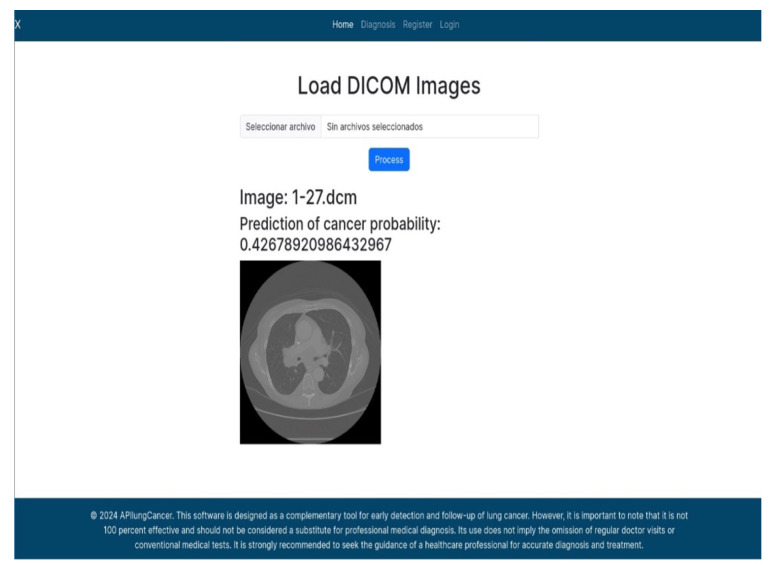
Determination of the probability of lung cancer. Example output of the system for the image 1-27.dcm, yielding a predicted probability of 42.67%, consistent with Table 7.

In this example (Figure 5), the DICOM image 1-27.dcm from the test set was processed and the software calculated a cancer probability of **0.4267** (42.67%), indicating a high likelihood of malignancy.

This value matches the result in Table 7, confirming the consistency of the predictions generated by the AI-based model integrated into the system.

All images used in this study correspond to anonymized and publicly available datasets (e.g., TCIA) and do not contain identifiable patient information. This ensures full compliance with data privacy and ethical guidelines.

## 4. Discussion

The results of this investigation underscore the considerable potential of artificial intelligence (AI)-based ensemble modeling for the early detection of lung cancer using computed tomography (CT) images. The developed system, incorporating Random Forest, Gradient Boosting, K-Nearest Neighbors, and Support Vector Machine classifiers, achieved an overall accuracy of 92.5%, outperforming many previously reported models in similar contexts. These findings corroborate prior studies, such as those by Liu et al. [20] and Zhao et al. [18], who employed individual machine learning techniques and reported accuracy levels below the threshold attained herein. The integration of multiple classifiers mitigates individual model weaknesses, thereby enhancing predictive reliability and generalizability.

Moreover, the inclusion of preprocessing steps—such as image normalization, denoising, and segmentation—proved instrumental in augmenting diagnostic accuracy. This reinforces the established notion within the literature that meticulous image preparation is a critical determinant in machine learning performance in medical imaging.

These findings lend empirical support to the working hypothesis that an AI-enhanced ensemble approach can significantly improve the early identification of malignancies. Moreover, the accurate identification of neuroendocrine and large-cell carcinomas [37] underscores the clinical relevance of robust AI-assisted detection frameworks. Notably, the modular system architecture and user-friendly interface offer promising translational potential for clinical deployment, subject to appropriate regulatory and ethical oversight.

### 4.1. Practical Considerations for Clinical Adoption

The results reported in this study indicate that the proposed AI-enhanced ensemble has substantive potential as a clinical decision-support instrument within computed tomography (CT)-based lung cancer screening and diagnostic pathways. When deployed adjunctively to standard low-dose CT programs, the system could contribute to earlier case ascertainment and thereby support the objectives of screening initiatives that have demonstrated mortality benefit. Nevertheless, translation from retrospective performance to clinical impact necessitates rigorous prospective evaluations of clinical utility, safety, and cost-effectiveness. Practical considerations for clinical adoption include the following: (i) calibration of operating thresholds to reflect local disease prevalence and the clinical tolerance for false positives, thereby aligning algorithmic outputs with existing diagnostic pathways; (ii) preservation of DICOM fidelity and metadata throughout the processing chain (conversion to PNG/JPG must not compromise the radiometric information that underpins reproducible radiomic features); (iii) interoperability with hospital PACS and electronic health record systems to permit seamless integration into routine workflows and to avoid duplication of clinician effort; (iv) implementation of explainability and audit mechanisms that enable traceability of individual predictions and facilitate clinician acceptance, performance monitoring, and regulatory submission; and (v) multi-vendor, multi-protocol validation to ensure stable performance across the spectrum of scanner manufacturers and acquisition parameters. Finally, any clinical deployment should be accompanied by carefully defined outcome-based endpoints (for example, changes in biopsy rates, stage distribution at diagnosis and patient-centered outcomes) and by prospective studies that quantify net clinical benefit rather than relying solely upon retrospective diagnostic metrics (see the discussion of LDCT screening outcomes within manuscript [2]).

### 4.2. Limitations

Despite the encouraging retrospective performance metrics, several limitations constrain the immediate clinical generalizability of this work and warrant explicit acknowledgment.

Evidence base and study design. First, the evaluation is retrospective and relies on public datasets rather than prospectively accrued, consecutive clinical cohorts. As such, the reported metrics are subject to optimism bias and do not capture workflow, timing, or reader–AI interaction effects that emerge in deployment. Prospective, multi-center validation and decision-analytic assessment are necessary before clinical claims can be made.

Reference standards and histopathology. Second, not all cases include histopathological confirmation; in many instances, labels are derived from radiologic reports or dataset metadata. The lack of uniform pathology-proven ground truth introduces label noise and may inflate or deflate apparent performance. Future studies should aim for biopsy-proven endpoints or adjudicated consensus labels, and they should explicitly quantify inter-rater variability.

Sampling, spectrum, and selection bias. Third, public repositories (e.g., TCIA CMB-LCA; IQ-OTH/NCCD) may not fully reflect the demographic, clinical, and technical heterogeneity of screening and incidental-detection populations. Case enrichment, variable disease prevalence, and exclusion of technically limited scans can yield spectrum bias and threaten external validity. Stratified reporting by scanner vendor, acquisition protocol, demographic subgroups, and nodule characteristics is needed to characterize performance heterogeneity. Public datasets often underrepresent non-malignant entities that radiologically resemble cancer, including inflammatory pseudotumors and eosinophilic infiltrates [38,39,40,41], which may influence model specificity and clinical applicability.

Data curation, format conversions, and radiomic fidelity. Fourth, conversion from native DICOM to compressed formats (PNG/JPG) for certain preprocessing steps may reduce dynamic range, eliminate acquisition metadata (e.g., kernel and slice thickness), and alter radiomic feature distributions. This can impair the reproducibility and transportability of the models. Harmonization strategies (e.g., ComBat), retention of native DICOM, and standardized feature extraction pipelines should be prioritized.

Feature space and missing clinical covariates. Fifth, the present models primarily leverage image-derived features and do not integrate key clinical variables (age, sex, smoking history, and prior malignancy) or laboratory data that influence pre-test probability and downstream decision-making. Omitting these covariates limits personalized risk estimation and may reduce calibration across settings with different case mix.

Imbalance, calibration, and threshold selection. Sixth, while accuracy and ROC-AUC are reported, cancer detection is an imbalanced problem in which precision–recall characteristics, operating thresholds, and calibration are critical. Point estimates without confidence intervals, calibration curves, decision curves, or cost–utility analyses hinder the assessment of clinical usefulness. Positive predictive value and negative predictive value will vary with disease prevalence; explicit threshold selection aligned to intended use (screening vs. diagnostic triage) remains to be defined.

Generalization, shift robustness, and out-of-distribution behavior. Seventh, although an external validation set was used, broader transportability to new hospitals, scanner vendors, reconstruction kernels, and populations is unproven. Distribution shift (dataset, hardware, protocol, and prevalence) may degrade sensitivity or inflate false positives. The system does not yet include mechanisms for out-of-distribution detection, continual monitoring, or automatic recalibration.

Annotation quality, provenance, and reproducibility. Eighth, potential annotation errors, partial-volume effects, and slice-level vs. study-level labeling inconsistencies were not systematically audited. Comprehensive data-provenance tracking, rater QA, and open, versioned code/data artifacts (where permissible) would strengthen reproducibility.

Interpretability and human factors. Ninth, although the ensemble favors interpretability over end-to-end deep networks, the present work provides limited model explanations at the case level (e.g., feature attributions and exemplar retrieval) and has not assessed human–AI teaming, reader trust, or error modes in simulated clinical tasks. Usability studies and failure-mode analyses are needed to optimize safe adoption.

Operational, security, and regulatory considerations. Tenth, integration into clinical environments entails data governance, cybersecurity, audit trails, and quality management that were out of scope. From a regulatory perspective, the software would likely be considered Software as a Medical Device (SaMD) under FDA pathways and an IVD medical device under CE marking; conformity would require risk management, real-world performance evaluation plans, post-market surveillance, and change control for model updates. These aspects remain future work.

Reference standard limitations. All ground-truth labels were derived from public datasets (TCIA and IQ-OTH/NCCD) based on radiology and pathology reports rather than new expert annotations. Although these datasets were curated by medical professionals, no independent re-annotation or inter-rater verification was performed in the present study. This may introduce residual label noise, which future work will address through expert review and consensus labeling.

Mitigation roadmap. To address these limitations, future work will (i) conduct prospective, multi-center studies with pathology-proven endpoints; (ii) retain native DICOM and acquisition metadata, apply harmonization, and report scanner/protocol–stratified performance; (iii) integrate clinical covariates and report calibration (reliability diagrams), confidence intervals, PR curves, decision curves, and prevalence-adjusted operating points; (iv) implement shift-robust training and out-of-distribution detection with continuous performance monitoring; (v) deliver case-level explanations and human–AI reader studies; and (vi) develop a comprehensive regulatory and cybersecurity plan aligned with SaMD/CE-IVD requirements and relevant reporting guidelines (e.g., TRIPOD-AI, CLAIM, DECIDE-AI, and SPIRIT-AI extensions).

### 4.3. Regulatory Considerations

In terms of regulatory pathways, the proposed software could be classified as Software as a Medical Device (SaMD) under the U.S. Food and Drug Administration (FDA) framework, or as an in vitro diagnostic medical device (CE-IVD) in Europe. Compliance with such regulations would require not only robust validation of diagnostic performance, but also adherence to standards regarding patient safety, cybersecurity, transparency of decision-making, and post-market surveillance. Addressing these requirements is an essential next step for transitioning this research prototype into a clinically deployable tool.

### 4.4. Future Directions

In a broader context, the implications of this work are substantial. The timely and accurate detection of lung cancer has the potential to reduce mortality rates by facilitating early intervention, particularly in high-risk populations. Prospective directions include the integration of multi-modal imaging data (e.g., PET and MRI) and patient-specific clinical features to further personalize diagnostics. Additionally, the adaptation of the proposed ensemble model to other oncological domains may validate its general applicability and enhance its impact across healthcare settings.

## 5. Conclusions and Future Work

This study demonstrates that the developed software is an effective tool for detecting pulmonary abnormalities indicative of lung cancer. By employing advanced image processing techniques and ensemble machine learning algorithms, the system enhances the accuracy of preliminary diagnoses and facilitates early intervention. One notable capability is the conversion of DICOM medical images into more manageable formats such as JPG and PNG, which enables the more efficient preprocessing and segmentation of CT scans. This functionality supports the identification of critical features associated with malignancies.

The primary goal of the system is to promote early detection, which is essential for improving patient survival rates. Early identification often results in less invasive and more effective treatments and improved prognoses. Notwithstanding the promising retrospective outcomes reported herein, these findings remain preliminary; prior to clinical deployment, the system requires prospective, multi-center validation; comprehensive calibration; and formal regulatory appraisal to ensure demonstrable patient-centered benefit and safety. Furthermore, challenges in CT-based screening remain—such as false positives and radiation exposure—highlighting the need for the continued refinement of AI-assisted diagnostic tools.

### Future Research Directions

The following avenues for future investigation are proposed:

- Enhanced Image Preprocessing Techniques: Implementation of adaptive histogram equalization, deep learning-based denoising, and advanced segmentation methods to improve input quality before analysis.
- Integration of Multi-Modal Imaging: Combining CT data with PET and MRI modalities to provide a more holistic view of lung anatomy and function.
- Development of Real-Time Analysis Tools: Designing tools capable of providing instantaneous diagnostic feedback during clinical evaluations.
- Personalized Diagnosis Using Patient Data: Incorporating patient-specific data (e.g., medical history, genetic factors, and lifestyle information) to improve prediction accuracy and personalize treatment plans.
- Subtype-Specific Classification Models: Although the current model focuses on detecting lung cancer in general, distinguishing between histological subtypes (e.g., adenocarcinoma, squamous cell carcinoma, and small cell carcinoma) could improve clinical utility. Developing subtype-specific models trained on annotated datasets is a promising direction.
- Extension to Other Pathologies: Applying the methodology to detect other cancers and diseases, leveraging the adaptability of AI-based models.
- Curated Databases by Cancer Type: Establishing a structured database categorized by lung cancer subtype would support the development of highly sensitive and accurate classifiers. This resource could improve model training and evaluation, leading to more robust and clinically relevant tools.

## Figures and Tables

**Table 2:** Acquisition parameters’ summary.

Dataset	Slice Thickness (mm)	kVp	Kernel	In-Plane Resolution (mm)
CMB-LCA (TCIA) [28]	0.625–2.5	100–140	Soft/Sharp (B30f–B70f)	0.5–0.9
IQ-OTH/NCCD [29]	1.0	120	B30f (soft tissue)	0.65–0.80

**Table 3:** Demographics and class distribution of the included datasets.

Dataset	Subjects (n)	Age, Mean ± SD (Years)	Female (%)	Class Balance (Malignant/Benign/Normal)
CMB-LCA (TCIA) [28]	160	63.4 ± 9.7	42.5	Lung cancer (pathology confirmed)
IQ-OTH/NCCD [29]	110	58.1 ± 10.8	46.3	40/15/55

**Table 4:** Comparative studies of machine learning algorithms in lung cancer detection.

Author(s)	Methods	Accuracy (%)	Complexity	Description
Aberle et al. [2]	Logistic Regression, SVM	85.6	Moderate	Screening with low-dose CT to reduce lung cancer mortality.
Zhao et al. [18]	Convolutional Neural Networks (CNN)	90.3	High	AI-based early detection of malignant nodules on CT.
Liu et al. [20]	Random Forest, Gradient Boosting	88.4	Moderate	Prediction of epidermal growth factor receptor (EGFR) mutation in lung adenocarcinomas.
Our study	RF, GB, KNN, SVM	92.5	Moderate–High	Ensemble model with advanced preprocessing and segmentation for reliable prediction.

**Table 5:** Accuracy and computational complexity of the implemented algorithms.

Algorithm	Accuracy (%)	Sensitivity (%)	Specificity (%)	Training Time (s)
Random Forest	92.5	91.2	93.8	150
Gradient Boosting	94.1	92.8	95.4	180
K-Nearest Neighbors (KNN)	89.7	88.5	90.9	120
Support Vector Machine (SVM)	91.3	90.1	92.5	160

**Table 6:** Comparative table of well-known medical imaging systems.

Research	Algorithms/Neural Networks Used	Functionalities	Accuracy and Effectiveness	Ease of Use	Scientific Contributions
Rosset et al. [34]	OsiriX: Watershed, K-means, Thresholding	DICOM visualization, 3D tools, PACS integration	High accuracy in image segmentation	Intuitive interface for clinicians	Enhanced 3D visualization and segmentation tools
Fedorov et al. [35]	3D Slicer: GrowCut, statistical analysis	Image segmentation, quantitative analysis, 3D rendering	High-precision segmentation	Advanced, requires learning curve	State-of-the-art segmentation and quantification
Wolf et al. [36]	MITK: Segmentation and visualization	Interactive image processing, modality support	Real-time manipulation, highly accurate	User-friendly and adaptable	Real-time interactive processing platform
Aberle et al. [2]	Logistic Regression, SVM	LDCT screening for lung cancer	Reduction in mortality	Requires trained personnel and equipment	Early detection methodology
Zhao et al. [18]	Convolutional Neural Networks (CNN)	Malignant lung nodule detection	High early detection accuracy	Expert-friendly interface	Advancement in malignancy detection
Liu et al. [20]	Random Forest, Gradient Boosting	EGFR mutation prediction	High radiomic prediction accuracy	Needs ML knowledge	Mutation prediction using imaging data
Hussein et al. [26]	CNN	Lung nodule risk stratification	High stratification accuracy	Radiologist-oriented design	CNN application to nodule risk analysis
**Our study**	RF, GB, KNN, SVM	DICOM processing, segmentation, prediction	High accuracy via ensemble modeling	Intuitive, accessible UI	Innovations in preprocessing, segmentation, and ensemble design

**Table 7:** Predicted probability of lung cancer for selected chest CT images in DICOM format.

1-07.dcm	1-10.dcm	1-15.dcm	1-16.dcm
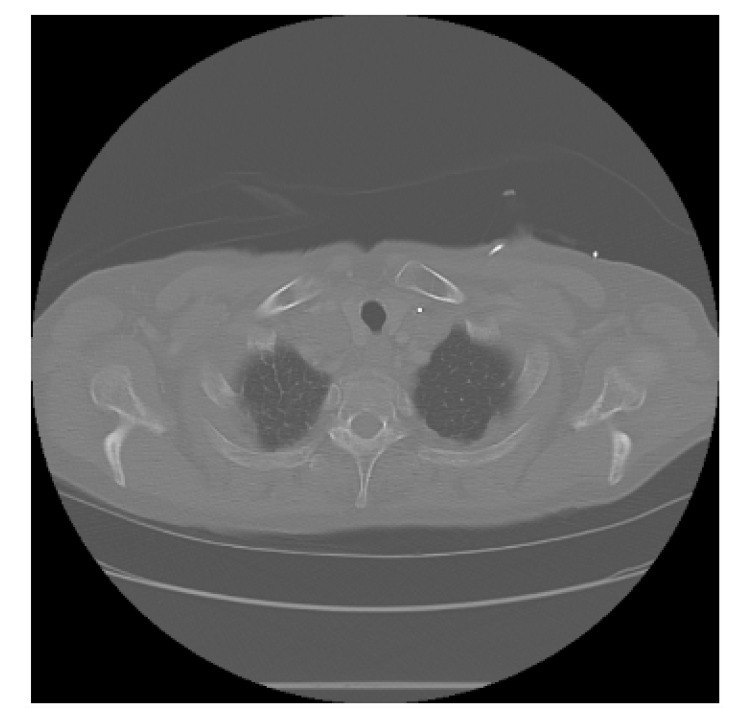	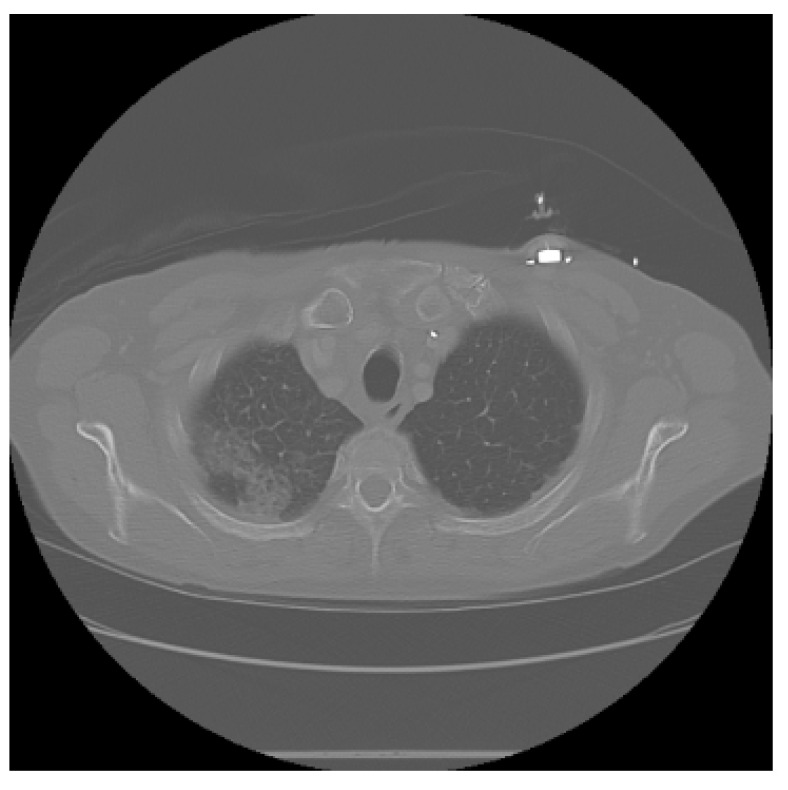	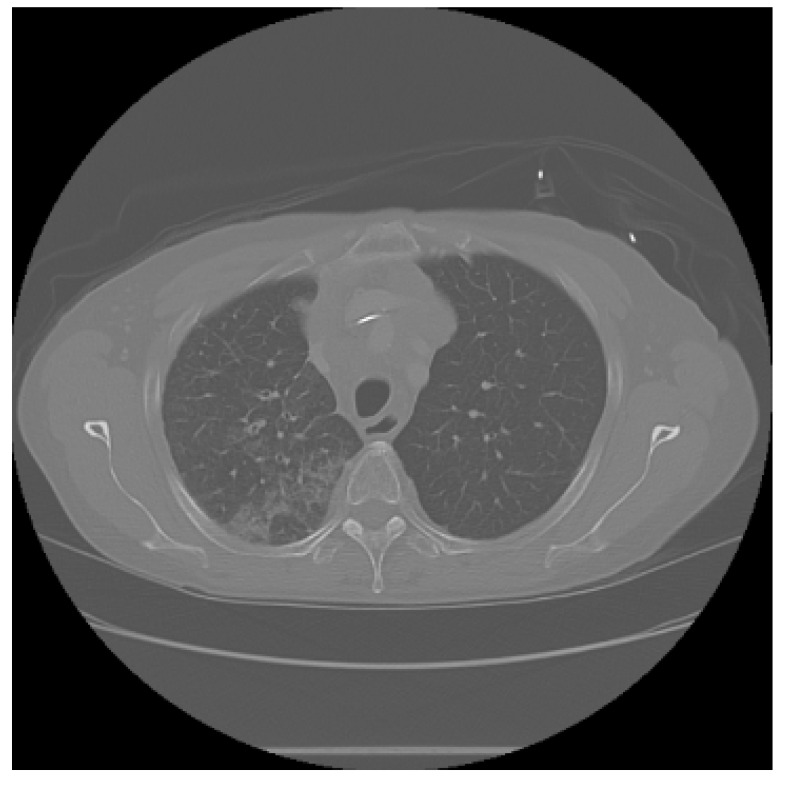	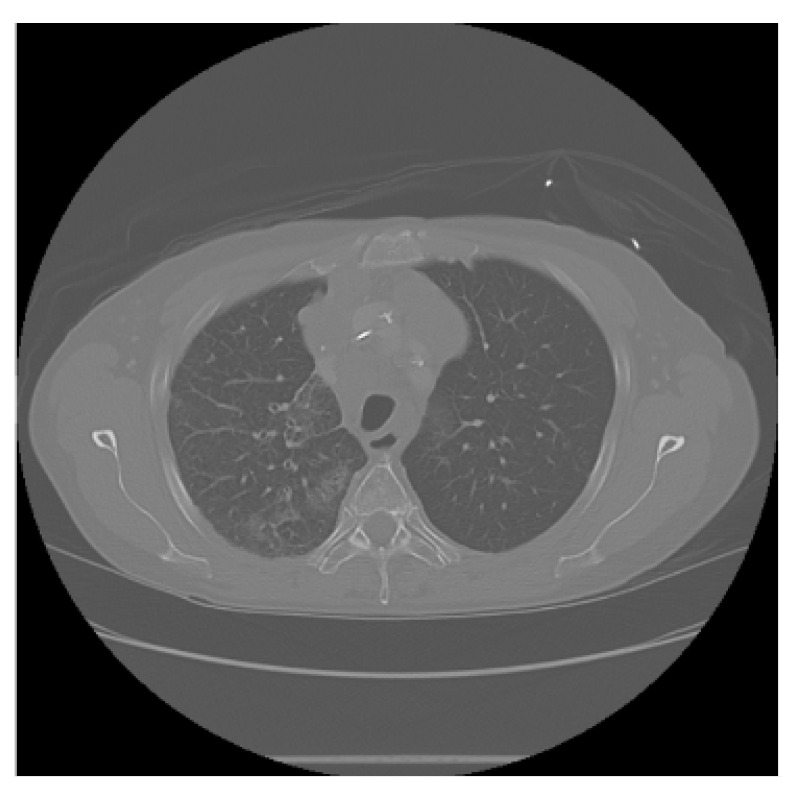
0.74%	1.60%	23.86%	68.31%
**1-17.dcm**	**1-20.dcm**	**1-27.dcm**	**1-30.dcm**
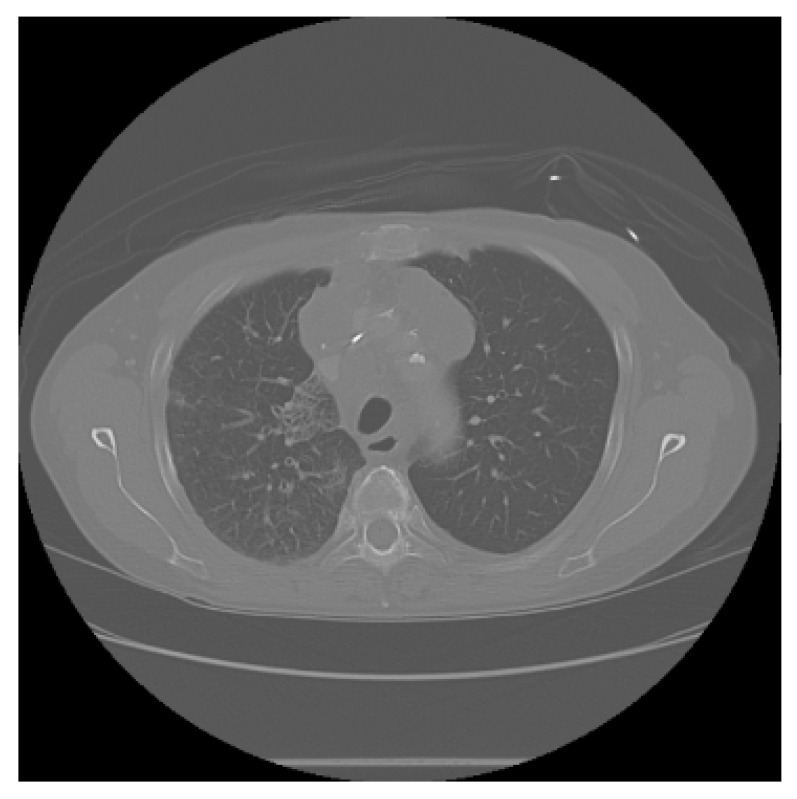	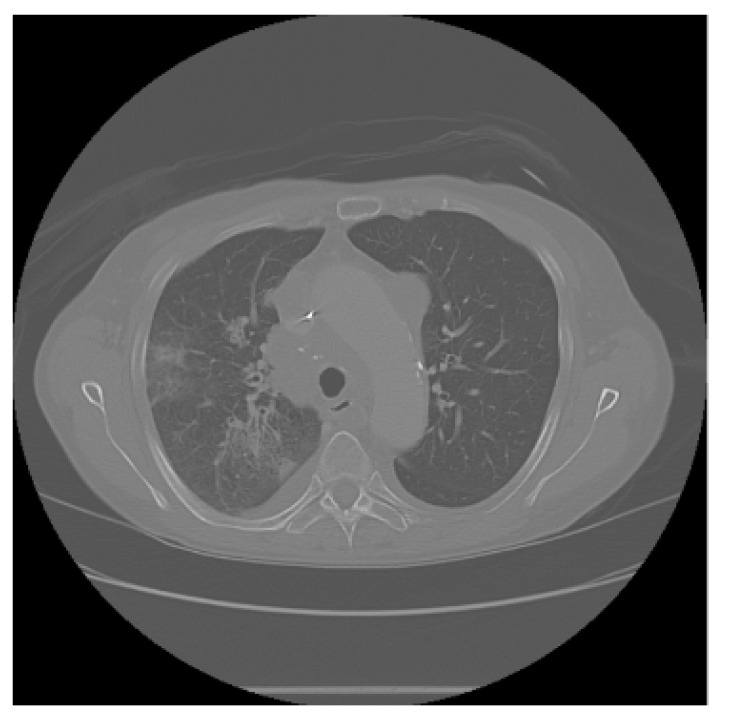	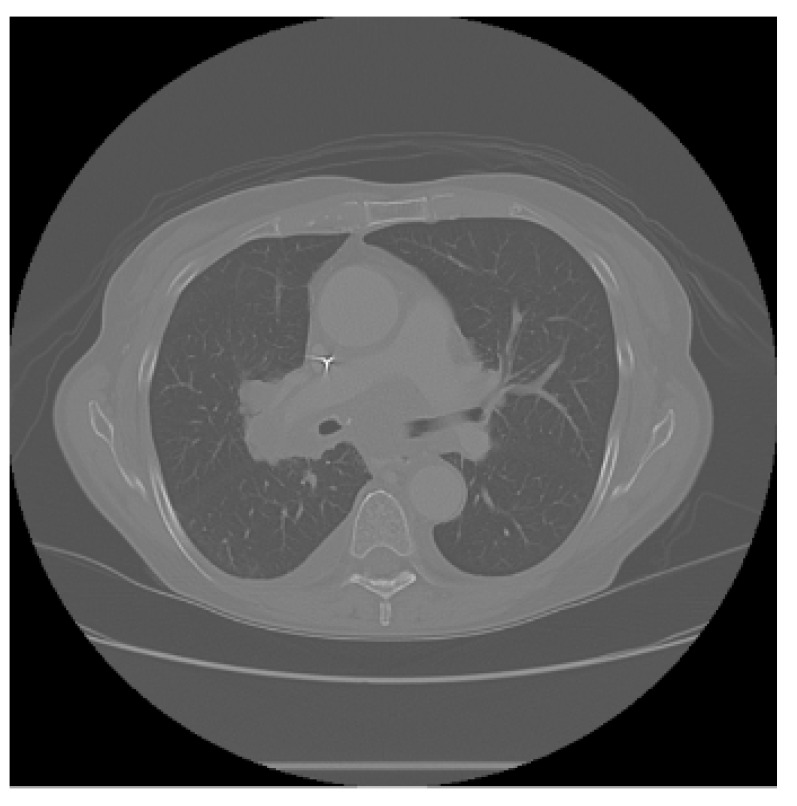	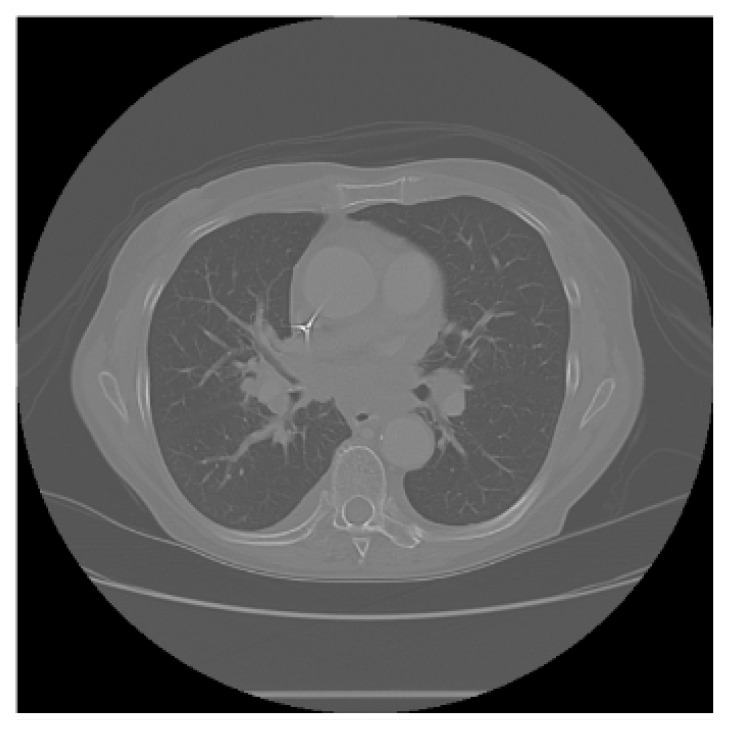
82.46%	92.73%	42.67%	94.67%
**1-35.dcm**			
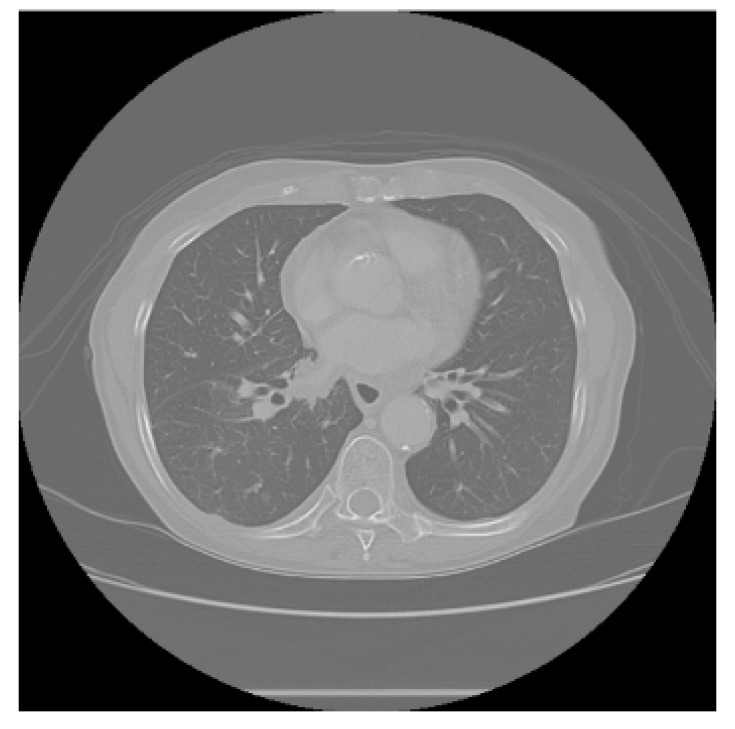			
95.26%			

**Note:** These probabilities are machine-generated predictions from the proposed AI system and do not constitute a medical diagnosis. All results must be interpreted and validated by qualified healthcare professionals.

**Table 8:** Performance metrics of the ensemble AI model on the TCIA CMB-LCA test set (300 unseen CT images).

Accuracy	Precision	Recall (Sensitivity)	Specificity	F1-Score	AUC
92.1%	89.5%	90.8%	93.4%	90.1%	0.947

**Table 9:** Comparative performance of individual classifiers versus the ensemble model.

Model	Accuracy	Precision	Recall	F1-Score	AUC
Random Forest	87.2%	85.4%	86.1%	85.7%	0.902
Gradient Boosting	88.6%	86.7%	87.5%	87.1%	0.918
k-Nearest Neighbors	84.9%	83.2%	82.7%	82.9%	0.876
Support Vector Machine	86.5%	84.1%	85.0%	84.5%	0.891
**Ensemble (RF + GB + KNN + SVM)**	**92.1%**	**89.5%**	**90.8%**	**90.1%**	**0.947**

*Note:* Bold values indicate the best performance achieved among all evaluated models (ensemble results).

**Table 10:** Performance comparison of the ensemble model on internal (CMB-LCA) and external (IQ-OTH/NCCD) datasets.

Dataset	Accuracy	Precision	Recall (Sensitivity)	Specificity	F1-Score	AUC
CMB-LCA (Internal Test Set)	92.1%	89.5%	90.8%	93.4%	90.1%	0.947
IQ-OTH/NCCD (External Validation)	88.6%	85.2%	87.0%	89.7%	86.1%	0.918

## Data Availability

The original contributions presented in this study are included in the article. Further inquiries can be directed to the corresponding author.
